# Capturing the Spectrum of Interaction Effects in Genetic Association Studies by Simulated Evaporative Cooling Network Analysis

**DOI:** 10.1371/journal.pgen.1000432

**Published:** 2009-03-20

**Authors:** Brett A. McKinney, James E. Crowe, Jingyu Guo, Dehua Tian

**Affiliations:** 1 Department of Genetics, University of Alabama School of Medicine, Birmingham, Alabama, United States of America; 2 Departments of Pediatrics, Microbiology and Immunology, Program in Vaccine Sciences, Vanderbilt University Medical Center, Nashville, Tennessee, United States of America; Wellcome Trust Sanger Institute, United Kingdom

## Abstract

Evidence from human genetic studies of several disorders suggests that interactions between alleles at multiple genes play an important role in influencing phenotypic expression. Analytical methods for identifying Mendelian disease genes are not appropriate when applied to common multigenic diseases, because such methods investigate association with the phenotype only one genetic locus at a time. New strategies are needed that can capture the spectrum of genetic effects, from Mendelian to multifactorial epistasis. Random Forests (RF) and Relief-F are two powerful machine-learning methods that have been studied as filters for genetic case-control data due to their ability to account for the context of alleles at multiple genes when scoring the relevance of individual genetic variants to the phenotype. However, when variants interact strongly, the independence assumption of RF in the tree node-splitting criterion leads to diminished importance scores for relevant variants. Relief-F, on the other hand, was designed to detect strong interactions but is sensitive to large backgrounds of variants that are irrelevant to classification of the phenotype, which is an acute problem in genome-wide association studies. To overcome the weaknesses of these data mining approaches, we develop Evaporative Cooling (EC) feature selection, a flexible machine learning method that can integrate multiple importance scores while removing irrelevant genetic variants. To characterize detailed interactions, we construct a genetic-association interaction network (GAIN), whose edges quantify the synergy between variants with respect to the phenotype. We use simulation analysis to show that EC is able to identify a wide range of interaction effects in genetic association data. We apply the EC filter to a smallpox vaccine cohort study of single nucleotide polymorphisms (SNPs) and infer a GAIN for a collection of SNPs associated with adverse events. Our results suggest an important role for hubs in SNP disease susceptibility networks. The software is available at http://sites.google.com/site/McKinneyLab/software.

## Author Summary

Susceptibility to many diseases and disorders is caused by breakdown at multiple points in the genetic network. Each of these points of breakdown by itself may have a very modest effect on disease risk but the points may have a much stronger effect through statistical interactions with each other. Genome-wide association studies provide the opportunity to identify alleles at multiple loci that interact to influence phenotypic variation in common diseases and disorders. However, if each SNP is tested for association as though it were independent of the rest of the genome, then the full advantage of the variation from markers across the genome will be unfulfilled. In this study, we illustrate the utility of a new approach to high-dimensional genetic association analysis that treats the collection of SNPs as interacting on a system level. This approach uses a machine-learning filter followed by an information theoretic and graph theoretic approach to infer a phenotype-specific network of interacting SNPs.

## Introduction

Human genetics studies have been successful at identifying single-locus variants that have a large effect on Mendelian disorders, such as cystic fibrosis or neurofibromatosis. However, the analytical strategies appropriate for identifying Mendelian disease genes have been met with limited success when applied to common multigenic diseases [1],[2]. Contributing to this limited success is the fact that the Mendelian approach requires that each susceptibility factor exert a large independent (main) effect on disease risk because association with the phenotype is investigated only one genetic locus at a time. The complexity of molecular interactions necessary to regulate gene expression likely is reflected at the DNA sequence level in the form of statistical interactions between alleles, with many of the individual alleles having little or no main effect on disease risk. The breakdown in the buffering against complex disease-related changes in expression may only be observable if properly investigated in terms of statistical interactions between genetic variants like single nucleotide polymorphisms (SNPs) or copy number polymorphisms (CNPs). Thus, analytical strategies for genetic association studies are needed that identify conditionally-dependent (interacting) susceptibility factors in addition to factors that exhibit an independent effect. Such strategies must be able to capture the spectrum of Mendelian to multifactor interaction effects.

Gene–gene interaction is widely accepted in the field of statistical genetics as a significant challenge to understanding the genetic architecture of complex diseases [3]–[9]. There is empirical evidence from human studies and model organisms to suggest that gene–gene interactions contribute to variation in complex diseases [10]–[15]. In human studies, for example, interactions were detected in Alzheimer disease between GAB2 and APOE [16], and high-risk haplotypes displaying intralocus interactions were detected in exfoliation glaucoma [17] and atrial fibrillation [18]. Another notable example of the importance of interactions in human disease is Hirschsprung's disease which was found to be influenced by polymorphisms in RET and EDNRB in the Old Order Amish [19]. This association was confirmed in a mouse model and the synergistic effect of both variants greatly outweighed the additive risk of each variant when considered independently. Like the examples above, susceptibility to common diseases, such as cancer, diabetes, obesity, hypertension, and premature cardiovascular disease, is likely influenced by the interaction of SNPs in multiple genes. Moreover, Mendelian disorders display a wide range of phenotypic variation that may be explained by interactions of the primary mutation with genetic modifier variants.

Our working definition of gene–gene interaction is the conditional dependence between genetic variants that affects the classification of the phenotype. This definition is equivalent to the definition based on deviation from additivity in a multi-locus model of phenotypic variation. These interactions may vary from very weak, or nearly additive, to purely epistasic, where it is only possible to detect a susceptibility locus when considered jointly with one or more additional loci. An advantage of genome-wide association (GWA) studies is that information about conditionally dependent loci is more likely to be available for gene–gene interaction analysis. Unfortunately, these useful genotypes are embedded in a genome-wide sea of noise, or variants irrelevant to classification of the phenotype. Thus, the focus of this paper is to address these two challenges in GWA studies: 1) accounting for gene–gene interactions and main effects and 2) removing noise variants to obtain a subset of SNPs that are enriched for association with the phenotype.

Random Forests (RF) [20] is a powerful nonparametric method that has been successfully applied to genetic data [21]. An RF is a collection of decision tree classifiers in which each tree in the forest has been trained on a bootstrap sample of instances from the data and each split attribute is chosen from among a random subset of attributes. In data mining terminology, an attribute is a dataset feature or variant such as a SNP, and an instance refers to a sample or subject. Out-of-bag instances are used to estimate prediction error and importance of each attribute via permutation testing. If randomly permuting values of a particular attribute does not affect the predictive ability of trees on out-of-bag samples, then that attribute will be assigned a low importance score [5],[21]. RF has been targeted as a method for identifying interactions in genetic data because it takes into account the context of other attributes when scoring the relevance of individual genetic variants and it does not require the specification of a model [22]. However, when association of an attribute with the phenotype is caused by a pure interaction with another attribute, the RF importance score of the relevant attribute diminishes. This limited ability to identify interacting attributes is due to the independence assumption used during node splitting, which is determined by the Gini index. The resulting trees are built on the assumed independence of the split attribute conditional on the class because the Gini split selector measures the impurity of the class value (case or control) distribution before and after the split on the evaluated attribute (*e.g.*, SNP).

Recursive Elimination of Features-F (Relief-F) is a heuristic attribute quality measure that can identify important variants in data sets that include strong interactions. However, Relief-F is sensitive to the presence of noise attributes, which when added to the data set cause Relief-F scores of relevant variants to worsen [23]. This limitation is exacerbated in GWA studies in which most of the variants may be irrelevant to the given phenotype. To overcome the bias caused by the context of noise attributes, strategies are necessary that iteratively remove variables with the worst Relief-F scores and update the scores of the remaining variables [23],[24]. The authors in Ref. [24] applied such a strategy, called tuned Relief-F (TuRF), to simulated genetic association data and demonstrated increased power to identify interacting SNPs over Relief-F without backwards elimination. Recently, we used evaporative cooling (EC) to create a composite score from Relief-F and information gain (IG), thereby demonstrating greater power than iterative Relief-F to detect pure interactions, with markedly greater power observed when one of the interaction partners demonstrated a marginal main effect. In real genome-wide association data, one expects both interaction and main effects to be present. Thus, the motivation of the EC filter is to optimize the linear combination of complementary scores to detect the continuum of independent and interaction effects. The EC approach in the current study optimizes the coupling of the RF and Relief-F scores based on classification accuracy and the iterative removal of noise attributes to obtain a collection of SNPs enriched for relevance to the phenotype.

The development of EC as a machine learning method was motivated by information theory and the statistical thermodynamics of cooling a gas of atoms by evaporation [26]. Just as a balance is struck between low energy and high entropy to achieve equilibrium in a collection of atoms, EC feature selection balances independent and interaction effects to obtain a collection of attributes enriched for association with the phenotype. Further, EC of a physical gas increases the phase space density by the iterative removal of the most energetic atoms while EC feature selection increases the feature space density by iteratively removing attributes that are least relevant to the phenotype. In a physical system, energy (*E*) and entropy (*S*) are balanced through the free energy *F* = *E*−*TS*, where *T* is the system temperature. In EC feature selection we optimize an analogous quantity that we call the information free energy, where E is the interaction contribution (Relief-F) and *S* is the main effect contribution. These two quantities are balanced by optimizing the coupling *T*. In our previous construction of the information free energy score we used IG, a quantity derived from information entropy, because the *S* contribution represents entropy in the thermodynamic free energy [26]. However, EC is not restricted to rely on an information entropy-based correlation score and, in fact, EC has a flexibility that allows it to couple any attribute quality scores. Thus, for the current study we use Relief-F as the interaction score and a transformation of the RF importance score as the independent effect score.

Through simulation analysis we show that the EC filter is able to identify genetic variants that confer risk through interaction with other genetic factors. Such risk factors may go undetected in a typical GWA analysis that reports a stringent list of the most significant SNPs where each SNP has been treated as independent. We apply the EC interaction filter to a real data set, which we analyzed previously for main effects using logistic regression (LR) [27]. The data set consists of 1442 SNPs across 386 candidate genes for subjects with and without systemic adverse events following smallpox vaccination. In order to characterize the interactions among the top EC-ranked SNPs, we infer what we call a genetic-association interaction network (GAIN). GAIN is based on interaction information (II), which was formulated by McGill [28] to quantify higher-order interaction gains between attributes and the class or phenotype. Jakulin and Bratko in Ref. [29] proposed a number of novel diagrams to visualize these interactions, some of which were incorporated by [30] into a strategy to characterize epistasis in multifactor dimensionality reduction (MDR) models. Positive connection strength between SNPs in a GAIN represents synergy between the two SNPs whose joint variation leads to improved classification of the phenotype. A negative network connection indicates redundant information between the two SNPs. In the terminology of genetics, “synergy” maps onto epistasis, and “redundancy” is most closely related to linkage disequilibrium but conditional on the phenotype. The EC filter, with its ability to select SNPs that may involve interactions or main effects, combined with GAIN for visualization and interpretation of the resulting network, provides an alternative approach to analyzing genotypic data on a more global scale, which will become increasingly important as GWA studies become more prevalent.

## Results

### Simulation Analysis

**Figure 1:**
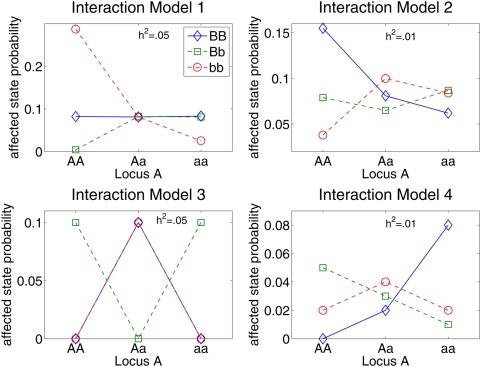
Penetrance diagram for genetic models used to compare strategies for filtering SNPs from case-control data. All four genetic models involve two interacting loci. Each point in a diagram is the probability of an individual being in the affected phenotype state for the given genotype combination. Model 3 is the purely epistatic XOR model, which displays no independent effects. The other interaction models display a small main effect through locus B. The models in the left column have .05 heritability and the models in the right column have .01 heritability. Analysis results for these simulated models are given in Figure 2.

Figure 1 depicts the two-locus interaction models simulated in this study to compare the performance of EC, TuRF, RF, and stepwise penalized LR (stepPLR) [31]. The models in Figure 1 include combinations of low heritabilities (h^2^ = .05 on the left and .01 on the right) and a range of interaction strengths (from nearly additive to completely epistatic). The 1% level represents a worst-case scenario for heritability, and the purely epistatic XOR model (Model 3) represents the worst-case scenario for gene–gene interaction models. For each genetic model, 100 replicate datasets were created with 1000 samples consisting of a balanced number of cases and controls. The proportions of the susceptibility alleles A and B in the population are assumed to be the same as the alleles a and b, respectively. Replicate simulations were created using the genomeSim software [32]. In addition to simulating the specified interaction model, genomeSim also simulates linkage disequilibrium (LD) patterns, though LD is not the focus of the current study. Each replicate dataset consists of a set of 1500 SNPs containing the two susceptibility SNPs.

**Figure 2:**
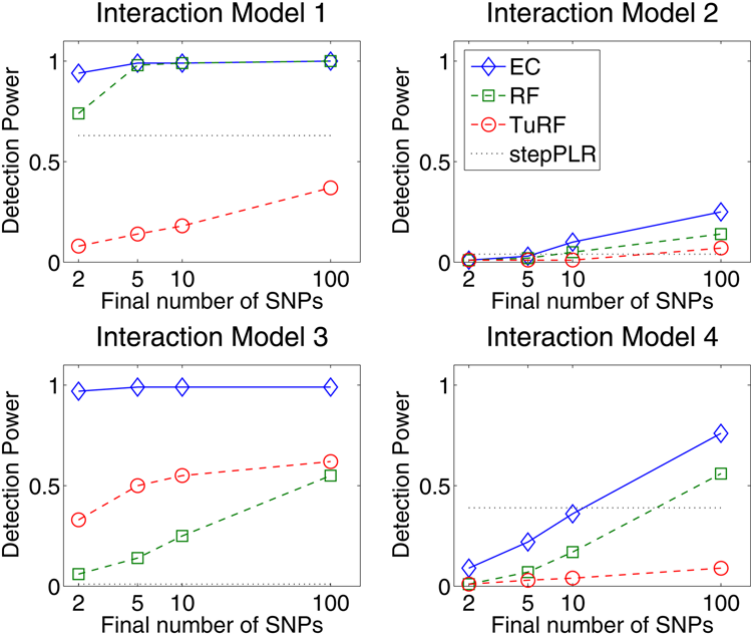
Detection power comparison of forward stepwise penalized logistic regression (stepPLR), Random Forests (RF), tuned Relief-F (TuRF) and Evaporative Cooling (EC) filter for 100 simulated replicates of each of the models defined in Figure 1 using 500 cases and 500 controls and 1500 total SNPs. Detection power is defined as the percentage of replicates for which both causal SNPs are found above the cutoff in the filter's rank list. The power is plotted as a function of the rank-list cutoff on a log scale, from the top 2 up to the top 100 final SNPs. EC, RF and TuRF results are sorted by importance score. Detection was counted for stepPLR if both causal SNPs had nonzero coefficients in the LR models.

Figure 2 summarizes the comparison of the ability of EC, TuRF, RF, and stepPLR to detect two-locus models described in Figure 1. For the 100 replicates of each model in Figure 1, we recorded the number of times that the two susceptibility SNPs were detected among the top filtered SNPs for each analytical method. The empirical detection power in Figure 2 is defined as the fraction of times out of all 100 replicate data sets for a given model that both of the simulated susceptibility SNPs occurred in the top SNPs as ranked by the given method. The cutoff for how many SNPs to include from the rank-list is varied in Figure 2 from the top 2 to the top 100 SNPs. In a real data analysis one may choose a filter cutoff that is larger than the top 2 because findings that replicate in multistage study designs often are not the most statistically significant associations in the initial scan [33],[34]. And, as we show below, a larger collection of SNPs permits a pathway-level analysis in which SNPs in multiple genes contribute to disease risk. Below we illustrate on a real data set a random permutation approach for selecting a significant EC cutoff score. When determining the top SNPs for EC, RF and TuRF, we sort by importance score. Detection is counted for stepPLR if both causal SNPs have a nonzero coefficient anywhere in the LR model. For RF, we used 10,000 trees in a forest and the square root of the total number of SNPs as the number of SNPs chosen randomly for node splitting. We used 10 nearest neighbors in the Relief-F calculations. For both RF and Relief-F, we used iterative removal of irrelevant SNPs in order to compare with EC consistently.

**Figure 3:**
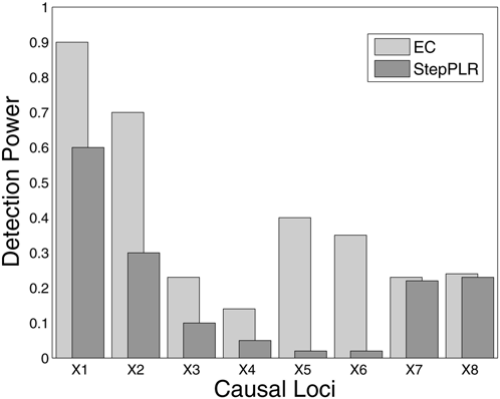
Detection power analysis of 100 simulated replicates of an 8-locus model that combines all genetic models defined in Figure 1. Each replicate has a population size of 500 cases and 500 controls and the eight functional loci are embedded in 1500 SNPs. Detection frequency is shown for each of the eight functional loci for EC with a cutoff of 50 SNPs compared with forward stepwise penalized logistic regression (StepPLR). The first two simulated loci (X1 and X2) follow the disease model defined in Interaction Model 1, the second two simulated loci (X3 and X4) come from Interaction Model 2 and so on.

The simulation results (Figure 2) show that EC does as well as, or improves upon, RF for all interaction models. For the interaction models with a small main effect (Models 1, 2, and 4), EC and RF perform similarly. For the interaction Model 2 with a small main effect and low (1%) heritability, EC and RF display modest power in the 20–25% range, while the power of TuRF is even lower at 7%. The weakness of RF at identifying purely epistatic models is most evident for Model 3, which has a relatively high (5%) heritability but is an XOR model with zero marginal effect. When the final number of SNPs is two, RF has only a 14% detection power for Model 3 whereas EC detects it with 91% power. For the interaction Model 2 with a non-vanishing main effect, all methods perform poorly due to the low (1%) heritability. It is likely that any analytical method would need a larger sample size to detect Model 2 with appreciable power. StepPLR shows a constant power for all models because of the small regularization parameter chosen by cross validation, which leads to models with fewer variables. For Model 4, StepPLR has an advantage over other methods when restricted to choosing the top two SNPs. EC performs better than TuRF for all models tested. TuRF shows its best performance for the XOR model, but performs worse than StepPLR for Models 1 and 4, which have an additive effect. By combining RF and Relief-F, the EC algorithm is able to detect interaction models with slight main effects, for which RF is well suited, and, leveraging the strength of Relief-F, EC is able to detect pure interaction models that RF is too myopic to detect. To illustrate the potential for detecting larger genetic models, Figure 3 shows the results of an analysis of 100 simulated replicates of an 8-locus model that combines the two-locus models from Figure 1. We compare the detection frequency for each of the eight functional loci for EC with a cutoff of 50 and StepPLR. This analysis shows the potential advantage of using EC as a filter for genetic models involving greater than two loci.

### Evaporative Cooling and Genetic-Association Interaction Network Analysis of Smallpox Vaccine Adverse Event Phenotype

To further validate and illustrate our approach, we apply the EC filter and GAIN strategy to a smallpox vaccine study looking at the association of a panel of SNPs with mild adverse events (AEs) [27]. Of the 131 subjects in the study, 40 experienced a systemic AE, which included fever, generalized rash and lymphadenopathy. Table 1 shows the top 26 SNPs out of the 1442 ranked by the EC filter for the vaccine AE phenotype. We arrived at this cutoff using the random permutation approach described in the Methods section. An EC score cutoff of −0.237 yields a .05 risk for a SNP that is declared relevant to the phenotype in Table 1 is actually irrelevant. As we dissect in more detail below, glycogen synthase kinase 3 beta (GSK3B) and solute carrier family 6 (neurotransmitter transporter, dopamine), member 3 (SLC6A3) in Table 1 are likely information hubs in this phenotype network. In addition to potential interaction effects, the EC relevance list also contains the same SNPs found in our previous main effect analysis in the 5,10-methylenetetrahydrofolate reductase (MTHFR) and interleuking-4 (IL4) genes.

**Table 1:** Top SNPs selected by Evaporative Cooling (EC) as most relevant to smallpox vaccine-associated adverse events.

SNP ID	dbSNP ids	Gene Name	EC Score
ESR1-13	56525559	estrogen receptor 1	−1.551
HSD17B4-19	21184487	hydroxysteroid (17-beta) dehydrogenase 4	−1.422
GSK3B-01	26231979	glycogen synthase kinase 3 beta	−1.174
GSK3B-27	26255314	glycogen synthase kinase 3 beta	−1.167
CASR-06	28496245	calcium-sensing receptor	−0.727
ALOX5-15	3320405	arachidonate 5-lipoxygenase	−0.701
RXRA-03	208756	retinoid X receptor, alpha	−0.633
AHR-17	16862729	aryl hydrocarbon receptor	−0.627
CD4-03	6783008	CD4 molecule	−0.605
SCUBE2-02	7859381	signal peptide, CUB domain, EGF-like 2	−0.597
ARNT-23	1340390	aryl hydrocarbon receptor nuclear translocator	−0.562
LIPC-08	29629829	lipase, hepatic	−0.561
OPRD1-03	11986807	opioid receptor, delta 1	−0.538
GSK3B-04	26074026	glycogen synthase kinase 3 beta	−0.493
MTHFR-02	1801133	5,10-methylenetetrahydrofolate reductase (NADPH)	−0.487
GSK3B-07	26090649	glycogen synthase kinase 3 beta	−0.483
EXO1-02	6787940	exonuclease 1	−0.480
MTHFR-02-2	6393745	5,10-methylenetetrahydrofolate reductase (NADPH)	−0.478
SLC6A3-14	1419969	solute carrier family 6 (neurotransmitter transporter, dopamine), member 3	−0.476
CYBB-12	488277	cytochrome b-245, beta polypeptide	−0.390
IL4-01	34424167	interleukin 4	−0.366
NFKB1-14	28084004	nuclear factor of kappa light polypeptide gene enhancer in B-cells 1	−0.321
IL4-03	34424723	interleukin 4	−0.312
CBR3-01	23169639	carbonyl reductase 3	−0.302
IL2-03	47925629	interleukin 2	−0.288
IL4-10	34433182	interleukin 4	−0.238

The EC score cutoff was selected by random permutation analysis, yielding a .05 risk that a SNP declared relevant on this list is actually irrelevant to the phenotype. SNPs sorted from best to worst EC score; a lower score means more relevance to the phenotype. SNPs are named according to their SNP500Cancer id (http://snp500cancer.nci.nih.gov/) in the first column and by dbSNP number (build 129) in the second column.

**Figure 4:**
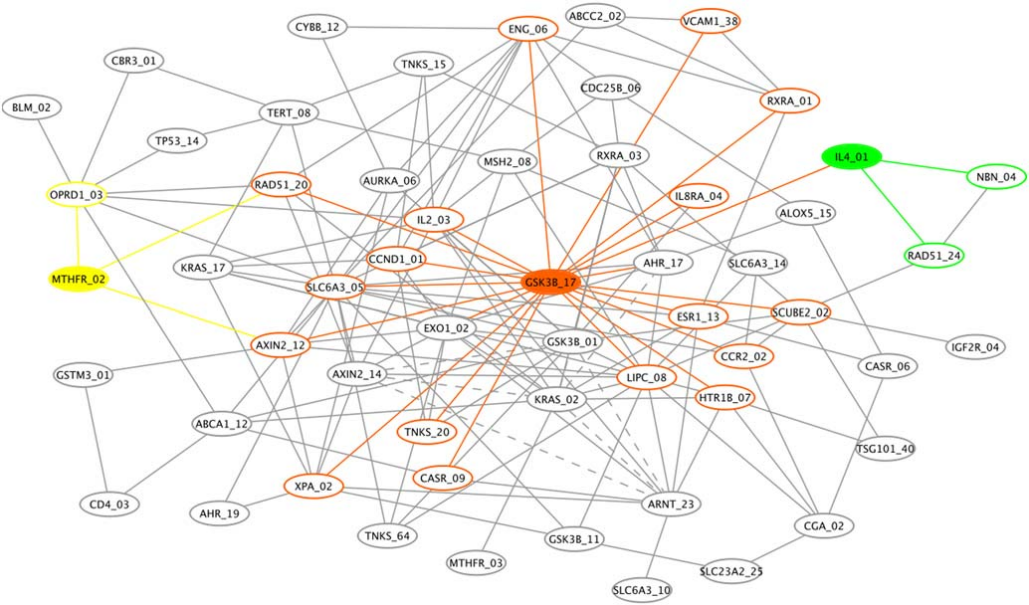
Genetic association interaction network (GAIN) for the top 70 SNPs selected by Evaporative Cooling (EC) as most relevant to smallpox vaccine-associated adverse events. Three nodes and their connections are highlighted: the hub glycogen synthase kinase 3 beta (GSK3B, orange) and the two main effect genes, 5,10-methylenetetrahydrofolate reductase (MTHFR, yellow) and interleukin-4 (IL4, green). Solid edges indicate synergy (positive interaction information (II)) between incident SNPs that results in an increase in information about the phenotype when the two SNPs are considered jointly. Dashed edges between incident SNPs indicate redundancy (negative II) with respect to information about the phenotype.

To characterize the details of the interaction network of this SNP-phenotype network, we draw upon the top 100 EC-ranked SNPs (see Supplementary Table 1), which we reduce to 70 SNPs by removing redundant markers. Specifically, if a pair of SNPs is correlated by more than .8 by the symmetric mutual information measure [26], then the least informative marker (lower EC score) is removed. The purpose of removing correlated SNPs is to reduce redundancy and make the network more interpretable. Another way to simplify the network would be to use nodes corresponding to constructed haplotypes. We also removed the least informative marker between pairs that are redundant in the context of the phenotype according to II. For example, GSK3B_01 and GSK3B_27 are correlated by less than .8; however, in the context of the phenotype they have nearly the same information content. Another way we plan to deal with correlated features in the future is to wrap an orthogonalization procedure into the EC method. Figure 4 shows the GAIN inferred from this EC-filtered list of SNPs.

The sample size of this vaccine trial is relatively small for identifying high-order interactions; thus, our goal in Figure 4 is simply to illustrate how the EC filter may be used in conjunction with GAIN to add insight into the network of interactions among SNPs that may influence the phenotype for a typical genetic association study. Edges represent interaction information (II) between SNPs, which does not simply represent correlation between SNPs but rather quantifies the amount by which their joint variation decreases our uncertainty about the phenotype over what would be expected by their individual effects (see Methods). For clarity of the graph, the number of connections displayed is limited to pairs of SNPs with the largest II magnitudes and to pairs involving nodes with the best EC scores. Specifically, we displayed edges between pairs of SNPs with an II magnitude greater than 6%, which results in 160 edges. The 6% II cutoff yields a .03 risk of obtaining a false connection, which was calculated by random permutation of SNP pairs (see Methods). Pairs of SNPs with positive II (synergy between the SNPs with respect to the phenotype) have solid edges. Pairs of SNPs with negative II (redundant information with respect to the phenotype) are indicated by dashed edges.

We highlight three nodes and their connections in Figure 4: a hub SNP in GSK3B (orange) and two main effect SNPs in MTHFR (yellow) and IL4 (green), which displayed main effect haplotypes in our primary analysis. GSK3B has a relatively large IG (see Supplementary Table 2 for numeric details of the GAIN interaction partners) but it may be more important in its influence on other SNPs in the context of the AE phenotype. For example, GSK3B has a direct connection with IL4 and a secondary connection with MTHFR. In the top ranking interaction pair (Suppl. Table 2), a SNP in GSK3B and lipase, hepatic (LIPC) contribute the most total gain in information about the phenotype despite the very small IG of LIPC. Supplementary Table 3 gives the connectivity distribution for the GAIN nodes. A SNP in SLC6A3 and GSK3B are hubs in the GAIN. Its synergy between several interaction partners and its independent effect give GSK3B one of the best EC scores (Table 1). The SNP in SLC6A3 has a much smaller main effect than GSK3B but its synergy leads to its having one of the better EC scores. Despite having a much smaller main effect, the SLC6A3 hub SNP appears to have an influential, though indirect, effect on the phenoytpe.

## Discussion

As pointed out by [35] an important challenge facing statistical genetics will be to balance the relative strengths and weaknesses of new and existing analytical methods because of the multiple challenges a single method must adequately address in a data set, including heterogeneity, gene–gene/gene-environment interactions, and genome-wide noise. The EC method attempts to achieve this balance via a machine learning optimization analogous to the way a system of particles achieves equilibrium by balancing low energy and high entropy as expressed through the thermodynamic free energy. The goal of EC is to address the challenge of disease model heterogeneity, gene–gene interactions, and noise by optimizing the coupling between two powerful machine learning/data mining methods with complementary strengths and weaknesses: Random Forest (RF) and Relief-F. The iterative removal of attributes (evaporation) plays a dual role by providing a mechanism for optimizing the coupling of RF and Relief-F and by removing attributes that are irrelevant to the phenotype (noise) from high-dimensional genotype data.

Relief-F was designed to account for interacting variants but consequently is more sensitive to noise. RF is more limited in its ability to identify interaction effects but is robust to noise, overfitting, and missing data. In addition, tree-based methods are suited to deal with certain types of genetic heterogeneity because splits near the root node define separate population subsets in the data. These methods exhibit complementary strengths and weaknesses. Thus, properly integrating these two scores and using backwards elimination allows EC to identify a spectrum of interaction effects, from purely epistatic XOR models to models displaying Mendelian effects. The selection from a random sample of attributes allows RF to maintain a low correlation between trees while the coupling with Relief-F by EC enriches the population of attributes for interaction effects that influence the phenotype.

Many SNPs in association studies have been shown to have small individual effect sizes, but their combined effect may be much larger. EC has high power to filter a large set of SNPs down to a small subset that is enriched for interaction and independent effects that influence association with the phenotype. The advantage of EC over standard statistical analysis is greatest when the genetic model contains no marginal main effect; however, EC performs as well as or better than RF for the interaction models that contain a main effect. EC also outperforms iterative Relief-F procedures (e.g., TuRF) with the greatest improvement occurring when one of the attributes demonstrates a small main effect. Thus, by balancing independent and interaction effects, EC is able to detect a spectrum of models in genetic association studies. Currently EC is meant to be an attribute filter for dimensionality reduction to be followed by more fine-grained modeling and/or a second stage of genotyping. However, in our simulation analysis we often find that the two functional SNPs have the highest EC rank, not just in a top percentile. In the application to real data we used a permutation approach to estimate an appropriate critical region of EC scores to reduce the number of false positive SNPs.

To characterize the genotype to phenotype map, we used the EC filter to reduce the space of SNPs to a more computationally manageable size for an exhaustive search for interactions by II, and then we used a network approach to visualize the interactions on a larger scale. Instead of using information theory to infer specific gene–gene interactions, one may use an alternative like pair-wise LR. The EC filter plus GAIN approach may prove to be a valuable complement to other approaches for modeling complex diseases because the inferred disease-specific network may better approximate the interconnectivity of the true biological system. In our second stage of analysis of the real data set, we inferred a genetic association interaction network (GAIN) in Figure 4, by taking advantage of the context dependence of all SNPs in the EC filter score. We again used a permutation strategy to prune the network by estimating the II cutoff appropriate for the given data. GAIN provides a visualization tool to explore, more globally, the statistical and biological relationships among the SNPs that are relevant to a given phenotype. GAIN is meant to be a discovery tool to suggest a SNP interaction network of the given phenotype. It provides information about synergy between SNPs whose joint effect increases information about the phenotype as well as information about redundancy between SNPs whose joint effect provides no additional information about the phenotype over their independent contributions.

The SNP hubs GSK3B, LIPC, and SLC6A3 (Supplementary Table 3) in Figure 4 have some of the highest EC scores and yield the largest information gains when joined with SNPs in other genes (Supplementary Table 2). Supplementary Table 2 only shows pair-wise interactions; however, GAIN Figure 4 suggests higher-order effects that can be decomposed into pair-wise interactions that cascade from the hub. The cascading effect of such hubs on disease susceptibility is an important area of investigation as is the identification of sub-networks in the GAIN that may suggest new pathways involving the given phenotype. As EC and GAIN are further developed it will be important to integrate gene ontology (GO) information with GAIN so that significantly enriched GO terms can be highlighted in network motifs. We used permutation to set the number of edges, but the use of prior knowledge may also help to determine the appropriate II cutoff magnitude for displaying GAIN edges, thereby reducing the number of false positive interactions in an inferred network.

For speed of analysis for large numbers of simulated data sets, this paper focused on candidate gene data sets on the order of 10^3^ SNPs but not high-density, whole genome data, which are typically on the order of 10^5^ or 10^6^ SNPs. To make whole-genome filtering feasible, we have implemented a version of EC that is parallelized (pEC). The freely available software for pEC results in a decrease in CPU time by a factor approximately equal to the number of processors used. Our strategy involves parallelizing the attribute quality evaluations (RF and Relief-F) in the evaporation loop since this is the most time consuming step of EC. We use a parallelized version of RF in Fortran 90, parallel RF (PARF) [36]. Using test data sets with a sample size of 1000 cases and 1000 controls, we estimate the computational speed of the current version of EC is 1.5 seconds per marker. Based on this rate, EC would be able to filter 1 million SNPs in 42 hours on 10 processors. The other computational advantage of EC over exhaustive search strategies is that EC takes into account the context of all SNPs when scoring a SNP, allowing for the inclusion of higher-order effects at no additional computational cost. Despite using a Naïve Bayes classifier to determine the parameter, *T*, for coupling importance score, EC shows very good power to identify interactions. We have tested other classifiers, such as decision trees, and have found little sensitivity to the choice of classifier. However, the method for optimizing the importance score coupling will be a focus of future research.

The genome-wide testing of DNA sequence variants for association with complex diseases opens up the possibility of identifying gene–gene interactions and even networks of interacting susceptibility loci. However, this network or pathway level view of SNPs affecting the expression of a phenotype will only be meaningful if analytical methods can identify gene–gene interactions. The EC filter is conducive to a pathway-level analysis because it accounts for the context of all SNPs when computing the relevance of a specific SNP to the phenotype. Furthermore, when coupled with network analysis such as GAIN, the collection of SNPs enriched for interactions may be modeled on a global/pathway level. We demonstrated the ability of EC network analysis to identify interactions between SNPs, the most common form of genetic variant, but EC is also applicable to gene expression data and the emerging CNP. By treating attributes as real-valued variables, gene expression data can be analyzed for interactive associations with a phenotype, and CNPs could be treated as discrete or real-valued to avoid converting raw intensities to genotypes. EC can be used for attribute selection in other domains of bioinformatics where statistical interactions may be significant, such as identifying biophysical properties of amino acids that predict protein binding.

## Methods

### Simulations

To compare the performance of each analytical method, replicate data sets for the genetic models in Figure 1 were created with the genomeSIM software package [32]. The genomeSIM software was developed as a realistic, forward-time population simulation algorithm that allows the user to specify many evolutionary parameters and to control evolutionary processes. In the simulation, an initial population of diploid individuals is randomly created and individuals cross by contributing one chromosome to the offspring. These crosses form the next generation and the process repeats until the specified number of generations has occurred. In the final generation, summing across the binary chromosome pairs at each position produces genotypes for the individual. Disease status is assigned by the probability of disease for each genotype or genotype combination as defined in the penetrance function.

### Stepwise Penalized Logistic Regression

Because LR is able to fit additive and other low order effects as well as interactions, we compare the filter methods in this paper with an LR with L_2_-regularization to fit gene interaction models [31]. As the number of markers in a genetic association data set grows it becomes increasingly unlikely that an exhaustive set of tests would be feasible, so a step-wise approach seems to be a reasonable approach for comparison with other filter methods. The authors in Ref. [31] implemented this method as an R package called stepPLR, which uses forward selection followed by backwards deletion for variable selection. In each forward step, a factor or interaction of factors is added to the model. In the backward step, factors are deleted beginning with the largest model from the forward steps. In our application, we selected the regularization parameter by cross-validation then built models based on the Bayesian information criterion.

### Relief-F

Relief-F is an extension of Relief, a heuristic machine learning method for estimating the quality of variants according to their ability to separate samples into classes. The following details of the algorithm apply to both Relief and Relief-F, then below we point out the differences. Consider a set of genetic variants (e.g., SNPs) *G*, where each genetic variant g_i_ in this set can be in one of the genotype states from the set *{0, 1, 2}*, corresponding to the homozygous for the common allele, heterozygous, and homozygous for the minor allele. In Relief, the weight of each attribute g_i_ is initially set to zero (W[g_i_] = 0) and for randomly selected samples (or for all samples if desired) the nearest hit and miss are computed with the chosen distance function (metric) and W[g_i_] is recursively updated according to how well the attribute can separate near hits and misses. Given a sample from one class, the nearest hit is defined as the nearest sample or individual from the same class as the sample of interest, where nearness in the SNP space is defined below. The nearest miss is the nearest sample from the opposite class. The selection of the *nearest* hit/miss is crucial to the success of Relief-F to find strong attribute dependencies because nearness is defined in the space of all SNPs as opposed to a single SNP at a time. For a given sample *S* (or individual) with nearest hit *H* and nearest miss *M*, the following equation is used to update the weight of each SNP g_i_:
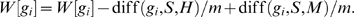
(2)


This is repeated for *m* samples selected randomly or exhaustively. Division by *m* in Eq. (2) ensures that the weight of each attribute lies between −1 and 1. For SNP g_i_, the difference function between samples S_j_ and S_k_ is
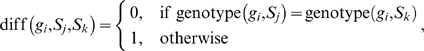
(3)where genotype(*g*,*S*) means the genotype of SNP *g* for sample *S*. Eq. (3) is used also for calculating the distance between samples to find the nearest neighbors. The total distance is the Manhattan distance, or the sum of distances over all SNPs.

The importance score *W* of a genetic variant *g_i_* is recursively updated for each individual, or sample *S*, in the population. Equation (2) rewards attributes that yield a large separation between the given sample and the nearest sample from the other class (misses, M) and penalizes attributes that give large separations between the given sample and the nearest sample from the same class (hits, H). For example, if the separation of a sample from its nearest hit is the same as its separation from its nearest miss then the contribution to the weight of the attribute is zero because it does not contribute to the classification of the sample. In our algorithm, we use Relief-F, an extension of Relief that enables it to handle noisy and incomplete data sets and to deal with multi-class problems. The main difference from Relief is that Relief-F searches for the K nearest hits and misses instead of the single nearest hit and miss, which gives greater robustness with respect to noise. We used K = 10 nearest neighbors and exhaustive selection of samples. For more details on Relief-F, see [37]. We use the Relief-F feature-weighting algorithm in our EC objective function (discussed below) because of its demonstrated ability to handle attribute interactions in genetic data [24],[26]. The iterative removal of the worst attributes in the evaporative formalism is the key to countering the devaluation of Relief-F importance scores of relevant SNPs due to the context of noise variants. As a control in the Results section, we compare EC with an iterative Relief-F called tuned Relief-F (TuRF) [24].

### Random Forests

In our original construction of EC, we used Information Gain (IG) as the main-effect contribution (the entropy term *S*) to the information free energy score [24],[26]. This was a natural choice for the evaporation formalism because of the basis of IG in information entropy. Although we show that Random Forest (RF) is not particularly good at identifying purely epistatic interactions (see Results), it performs very well when identifying main effect variants that elude many standard methods (e.g., IG, chi-square, LR). Thus, we integrate the Random Forest importance ranking as the main-effect component (*S*) to the EC score (discussed below). We use a version of RF known as PARF (parallel RF) that has been parallelized in Fortran 90 [36]. RF is a collection of decision tree classifiers, where each tree in the forest has been trained using a bootstrap sample of individuals from the data and each split attribute in the tree is chosen from among a random subset of attributes. Classification of individuals is based upon aggregate voting over all trees in the forest. Each tree in the forest is constructed as follows from data having *N* individuals and *M* explanatory attributes:

1. The method chooses a training sample by selecting *N* individuals with replacement from the entire dataset.
2. At each node in the tree, *m* attributes are selected randomly from the entire set of *M* attributes in the data. The absolute magnitude of *m* is a function of the number of attributes in the dataset and remains constant throughout the forest building process.
3. The method chooses the best split at the current node from among the subset of *m* attributes selected above.
4. The second and third steps are iterated until the tree is fully grown (no pruning).

Repetition of this algorithm yields a forest of trees, each of which has been trained on bootstrap samples of individuals. Thus, for a given tree, certain individuals were left out during training (on average for a large number of samples, the fraction 1-1/e). Prediction error and attribute importance was estimated from these “out-of-bag” individuals.

In RF the out-of-bag (unseen) individuals are used to estimate the importance of particular attributes according to the following logic: If randomly permuting values of a particular attribute does not affect the predictive ability of trees on out-of-bag samples, that attribute is assigned a low importance score. If, however, randomly permuting the values of a particular attribute drastically impairs the ability of trees to correctly predict the class of out-of-bag samples, then the importance score of that attribute is high. Tree methods are suited to dealing with certain types of genetic heterogeneity because splits near the root node define separate population subsets in the data. RF capitalizes on the established benefits of decision trees and has demonstrated excellent predictive performance when the forest is diverse (i.e., trees are not highly correlated with each other) and composed of individually strong classifier trees [20],[21].

By running out-of-bag samples down entire trees during the permutation procedure, weak attribute interactions are taken into account when calculating importance scores, since class was assigned in the context of other attribute nodes in the tree. However, RF has limited ability to identify strong interaction (pure epistatic) effects (see Results section). An approach for improving the ability of RF to identify interactions can be found in Ref. [25]. The author found a slight increase in the performance of RF when several attribute evaluation measures, including Relief-F, were used as the split selectors for building the trees instead of only the Gini index. Ref. [25] used classification accuracy as the performance measure, but in the current paper we are more interested in the power to identify specific genetic variants that predict the phenotype variable. Rather than integrate Relief-F into RF as the split selector, the EC approach used in the current study computes the RF importance score (with the Gini index) and computes the Relief-F score outside of RF then couples them into a composite importance score.

### Evaporative Cooling

**Figure 5:**
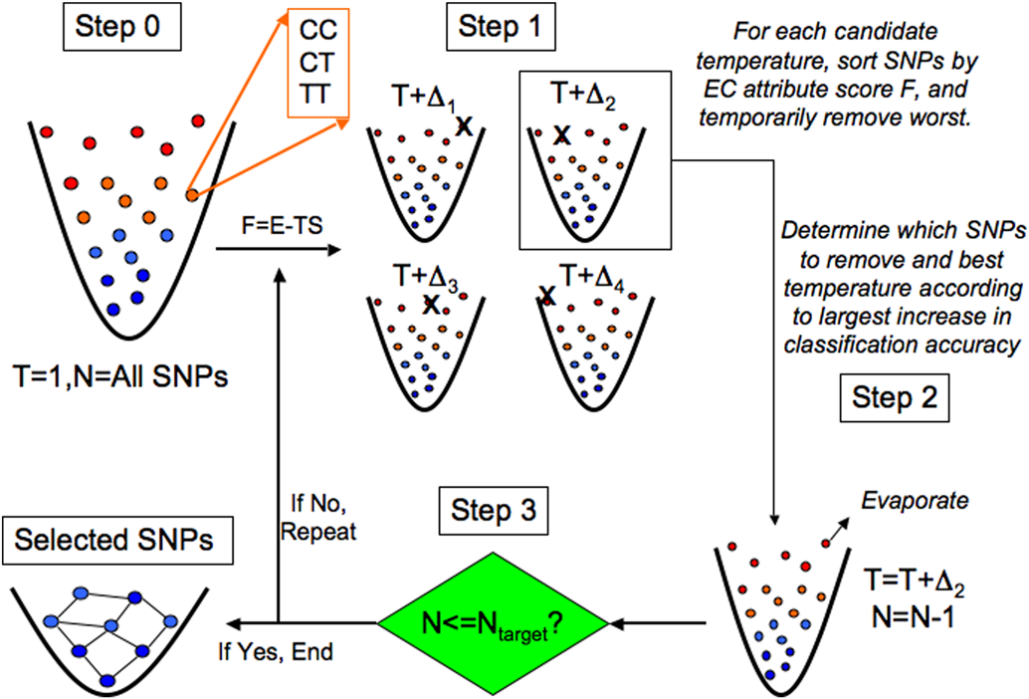
Evaporative Cooling (EC) feature selection algorithm. Each locus is conceptualized as a discrete-state particle with available states corresponding to its genotypes (e.g., CC, CT, TT) in a fictitious potential well, which controls the number of SNPs filtered. The information free energy *F* of each SNP is determined by its relevance to the phenotype. SNPs less relevant to the phenotype have higher free energy (more noise) and are positioned near the top of the potential well. Interaction (Relief-F, represented by *E*) and independent (Random Forest, represented by *S*) effect scores are coupled by the optimization parameter *T*, analogous to temperature in the free energy *F*. Initially, the information free energy *F* is calculated for all SNPs in the data set with the coupling constant *T = 1* (step 0). The coupling constant is varied about unity so that the set of SNPs is removed that gives the largest increase in classification accuracy over the previous iteration (step 1). This defines the updated coupling *T* and yields the new collection of SNPs with the SNPs evaporated with the most noise (least relevance to the phenotype) (step 2). If the target number of SNPs is reached (step 3), then a genetic-association interaction network (GAIN) is generated from the collection of SNPs that have been enriched for interactions and relevance to the phenotype by EC. Otherwise, if the target number of SNPs has not been reached yet, the coupling parameter again is varied about the previous coupling and the evaporation process is repeated. Permutation is used to select the target number of SNPs.

We introduced Evaporative Cooling (EC) as a machine learning method for feature selection in Ref. [26]. As illustrated in Figure 5, the heuristic used in our new EC machine-learning algorithm is the evaporation of a collection of atoms to reach equilibrium by balancing low energy (E) and high entropy (S) via the temperature (*T*) to minimize the free energy, *F = E-TS*. The physical process of evaporative cooling was first proposed as an experimental technique for cooling a small gas of atoms by [38]. The experimental method consists of the selective removal of atoms in the high-energy tail of the thermal distribution and the collisional equilibration of the remaining atoms. The combination of atom selection and collisions increases the phase-space density and can greatly reduce the temperature of a gas. In the EC machine learning analogy, each atom represents a variant with genotype states whereby each variant contributes quantities analogous to energy and entropy to the relevance to the phenotype. The orange highlighted SNP in Step 0 of Figure 5 has genotype states corresponding to homozygous for the C allele (CC), homozygous for the T allele (TT) and heterozygous (CT). Each SNP makes a contribution to the “information free energy,” *F = E-TS*, which quantifies the relevance of a collection of SNPs to the phenotypic variable. It is the goal of EC to minimize this quantity. The contributions to *F* of SNPs that are less relevant to the phenotype are positioned higher in the trap (parabola), and these SNPs are allowed to escape the trap as the top of the trap is lowered. The key mechanism of EC is the balance of statistical interactions (E) and independent effects (S) via the “information temperature” *T* as noisy variants (SNPs unrelated to the phenotype) are evaporated (iteratively filtered) from the full collection of SNPs in the trap, leaving behind a subset of SNPs enriched for relevance to the phenotype. An important advantage of the EC formalism is the ability to assimilate alternative SNP relevance scores through the coupling constant *T*. In the present study we couple RF and Relief-F to boost the performance over each attribute importance score alone.

Figure 5 gives an overview of the EC feature selection algorithm. At top left (Step 0), all *N* SNPs in the data set are represented as a gas of atoms in a fictitious trap, where more energetic SNPs (red, top) are poorly associated with the phenotype and “colder” SNPs (blue, bottom) are more closely associated with the phenotype. Relevance is determined by the attribute importance score *F = E-TS*, where *E* is the Relief-F score and *S* is the RF score, both transformed to be on the same scale with a range between 0 and 1. Relief-F is further transformed so that a SNP with a low Relief-F score is more important. The information temperature is initialized to *T = 1* (least biased assumption) so that the main effect and interaction terms of the attribute quality score are equally coupled. In Step 1, an ensemble of gases is created from the initial set of SNPs by variation D of the information temperature *T* around the initial value [we use the range D = *22*]. Since each collection in the ensemble uses a different coupling *T*+D, the rank order of the SNPs will differ in general. Thus, each collection of SNPs in the ensemble will have a different set of worst SNPs removed, indicated by an X in Step 1, corresponding to different perturbations D of the information temperature T. In Step 2 the new value of the temperature and the particular SNPs removed are determined by the collection of remaining SNPs generated in Step 1 that yield the highest classification accuracy. We use a naïve Bayes classifier as we have found little sensitivity to the type of classifier. The goal of Step 1 is to locally search for the information temperature that removes the worst attributes, and in Step 2 the worst SNPs are evaporated, or permanently removed.

In Step 3 the stopping criterion is checked. If the target number of SNPs (*N*_target_) has not been reached (“If No”), the evaporation procedure is repeated. In the example shown in Figure 5, the new temperature would become T = T+D_2_ from Step 2. Then iteration would continue with Step 1 with the SNPs ranked according to the new attribute importance score calculated by perturbing about this new temperature. The recalculation of *F* after the removal of noise attributes at each evaporation step is primarily motivated by the context dependence of Relief-F, which can lead to sensitivity to noise variants. However, we also find that RF benefits from the recalculation of importance scores. If on the other hand in Step 3 the number of SNPs is equal to or less than the target number of SNPs specified by the user (“If Yes”), then the stopping criterion has been met and the remaining SNPs become the final collection of “cooled” SNPs that are most relevant to the phenotype. This final collection of SNPs is depicted as a frozen network of interacting attributes, which is inferred as a genetic-association interaction network (GAIN) of the phenotype (discussed below). Just as evaporative cooling of an atomic gas increases the phase space density of the gas by repeatedly removing the most energetic atoms, the goal of EC feature selection is to alleviate the curse of dimensionality [39] by increasing the feature space density through the iterative removal of the genetic variants with the most noise. Relief-F makes the detection of interactions of order *m* computationally efficient because the complexity with respect to the number of SNPs, *n*, is O(*n*), versus O(*n^m^*) for an exhaustive search. The final number of SNPs, *N*_target_, is chosen based on a permutation strategy discussed below.

### Genetic-Association Interaction Network

The SNPs selected by EC are enriched for interactions as well as main effects, but EC does not predict which specific SNPs may be interacting. In order to characterize specific interactions among the top EC-ranked SNPs, we infer a genetic association interaction network (GAIN). GAIN is based on II [28] between three attributes (in this case, between two regular attributes *A* and *B* and the class attribute *C*):
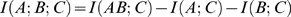
(4)where *I(A;C)* and *I(B;C)* are the information gained about the phenotype (C) when locus A and locus B, respectively, are measured. The quantity AB is a joint attribute constructed from attributes A and B with states given by the Cartesian product of the states of A and B. II is then the gain in class information obtained by considering A and B jointly beyond the class information that would be gained by considering variables A and B independently. We use the II (Eq. 4) as the connection strength of each edge in the GAIN (Figure 4). Thus, each edge represents the increase in information about the phenotype achieved by considering the two SNPs jointly compared to the expected increase in information with the assumption of independence between the SNPs. We emphasize that a connection between SNPs in a GAIN is specific to the given phenotype because it measures the correlation between two SNPs that influences association with the phenotype. We have made the Java software freely available for generating the GAIN results. We built network visualization into the software tool, but to create Figure 4 we used the export option in the GAIN software for subsequent visualization in Cytoscape [40], a freely distributed software tool for network visualization and annotation.

### Determining Statistical Thresholds by Random Permutation

A challenge for non-parametric methods like EC is assessing the statistical significance of a relevant SNP or, in the case of GAIN, a significant interaction between SNPs. We use a random permutation approach to determine a statistically significant threshold or cutoff for selecting the top EC SNPs and the top interaction pairs for GAIN. For EC we generate a distribution of irrelevant SNPs by randomly selecting SNPs with replacement and then calculating their EC score after randomly permuting the genotypes of the chosen SNP. From the resulting distribution of irrelevant SNPs, we determine the EC threshold by selecting the EC score such that only a fraction a of the irrelevant scores are more extreme. To select the interaction strength threshold for displaying GAIN edges, we calculate the II for randomly permuted pairs of SNPs. From the resulting non-interaction distribution of II scores, we use the same process to choose the threshold as we used for selecting the EC score threshold.

## Supporting Information

Table S1 Top 100 SNPs selected by Evaporative Cooling (EC) as most relevant to smallpox vaccine-associated adverse events. SNPs sorted from best to worst EC score. SNPs are named according to their SNP500Cancer id (http://snp500cancer.nci.nih.gov/) in the first column and by dbSNP number (build 129) in the second column.
(0.14 MB DOC)

Table S2 Pairs of SNPs ranked by their total joint information gain (last column), which is the sum of IG1, IG2, and pair-wise interaction information (II). IG1 (respectively, IG2) is the information gained about the phenotype variable when SNP interaction partner 1 (respectively, 2) is measured by itself. II (column 5) is the information gained about the phenotype when considering the SNP partners 1 and 2 jointly over what would be expected by their independent information gains. II is used to specify edge properties in Figure 3. Information gains are given as percentages, where perfect correlation with the phenotype is 100%. SNPs are named according to their SNP500Cancer id (http://snp500cancer.nci.nih.gov/).
(0.25 MB DOC)

Table S3 Degree distribution of nodes in the genetic-association interaction network (GAIN) in Figure 3 for the smallpox vaccine-associated adverse event phenotype. SNPs are sorted by the degree (number of connections) of each SNP node in the network. SNPs are named according to their SNP500Cancer id (http://snp500cancer.nci.nih.gov/). dbSNP numbers can be found in Table S1.
(0.07 MB DOC)
