# Supplementary material for: Capturing the Spectrum of Interaction Effects in Genetic Association Studies by Simulated Evaporative Cooling Network Analysis
Source: PLoS Genet. 2009 Mar 20;5(3):e1000432. doi: 10.1371/journal.pgen.1000432 (PMC2653647; doi:10.1371/journal.pgen.1000432)
Supplement: Table S1 — Top 100 SNPs selected by Evaporative Cooling (EC) as most relevant to smallpox vaccine-associated adverse events. SNPs sorted from best to worst EC score. SNPs are named according to their SNP500Cancer id (http://snp500cancer.nci.nih.gov/) in the first column and by dbSNP number (build 129) in the second column. (0.14 MB DOC) [file pgen.1000432.s001.doc]

| **SNP ID** | **dbSNP ids** | **Gene Name** | **EC Score** |
| --- | --- | --- | --- |
| ESR1-13 | 56525559 | estrogen receptor 1 | -1.551 |
| HSD17B4-19 | 21184487 | hydroxysteroid (17-beta) dehydrogenase 4 | -1.422 |
| GSK3B-01 | 26231979 | glycogen synthase kinase 3 beta | -1.174 |
| GSK3B-27 | 26255314 | glycogen synthase kinase 3 beta | -1.167 |
| CASR-06 | 28496245 | calcium-sensing receptor | -0.727 |
| ALOX5-15 | 3320405 | arachidonate 5-lipoxygenase | -0.701 |
| RXRA-03 | 208756 | retinoid X receptor, alpha | -0.633 |
| AHR-17 | 16862729 | aryl hydrocarbon receptor | -0.627 |
| CD4-03 | 6783008 | CD4 molecule | -0.605 |
| SCUBE2-02 | 7859381 | signal peptide, CUB domain, EGF-like 2 | -0.597 |
| ARNT-23 | 1340390 | aryl hydrocarbon receptor nuclear translocator | -0.562 |
| LIPC-08 | 29629829 | lipase, hepatic | -0.561 |
| OPRD1-03 | 11986807 | opioid receptor, delta 1 | -0.538 |
| GSK3B-04 | 26074026 | glycogen synthase kinase 3 beta | -0.493 |
| MTHFR-02 | 1801133 | 5,10-methylenetetrahydrofolate reductase (NADPH) | -0.487 |
| GSK3B-07 | 26090649 | glycogen synthase kinase 3 beta | -0.483 |
| EXO1-02 | 6787940 | exonuclease 1 | -0.480 |
| MTHFR-02-2 | 6393745 | 5,10-methylenetetrahydrofolate reductase (NADPH) | -0.478 |
| SLC6A3-14 | 1419969 | solute carrier family 6 (neurotransmitter transporter, dopamine), member 3 | -0.476 |
| CYBB-12 | 488277 | cytochrome b-245, beta polypeptide | -0.390 |
| IL4-01 | 34424167 | interleukin 4 | -0.366 |
| NFKB1-14 | 28084004 | nuclear factor of kappa light polypeptide gene enhancer in B-cells 1 | -0.321 |
| IL4-03 | 34424723 | interleukin 4 | -0.312 |
| CBR3-01 | 23169639 | carbonyl reductase 3 | -0.302 |
| IL2-03 | 47925629 | interleukin 2 | -0.288 |
| IL4-10 | 34433182 | interleukin 4 | -0.238 |
| IL4-11 | 34428976 | interleukin 4 | 0.032 |
| MTRR-22 | 7879216 | 5-methyltetrahydrofolate-homocysteine methyltransferase reductase | 0.032 |
| MTRR-05 | 7879304 | 5-methyltetrahydrofolate-homocysteine methyltransferase reductase | 0.044 |
| BLM-02 | 6255893 | Bloom syndrome | 0.068 |
| AURKA-02 | 1047972 | serine/threonine-protein kinase 6 | 0.070 |
| LTA-05 | 22398393 | lymphotoxin alpha (TNF superfamily, member 1) | 0.089 |
| XPA-02 | 7780783 | xeroderma pigmentosum, complementation group A | 0.122 |
| GSK3B-12 | 26304751 | glycogen synthase kinase 3 beta | 0.203 |
| GSK3B-40 | 26237867 | glycogen synthase kinase 3 beta | 0.208 |
| GSK3B-42 | 26244557 | glycogen synthase kinase 3 beta | 0.213 |
| SLC6A3-05 | 1436389 | solute carrier family 6 (neurotransmitter transporter, dopamine), member 3 | 0.215 |
| TSG101-36 | 17335787 | tumor susceptibility gene 101 | 0.215 |
| KRAS-02 | 18155009 | Kirsten rat sarcoma viral oncogene homolog | 0.236 |
| RAD51-16 | 11797080 | RAD51 homolog (RecA homolog, E. coli) (S. cerevisiae) | 0.242 |
| GSK3B-28 | 26266417 | glycogen synthase kinase 3 beta | 0.244 |
| TNKS-20 | 2055337 | tankyrase, TRF1-interacting ankyrin-related ADP-ribose polymerase | 0.247 |
| TNKS-110 | 2055094 | tankyrase, TRF1-interacting ankyrin-related ADP-ribose polymerase | 0.248 |
| IL8RA-04 | 69236919 | interleukin 8 receptor, alpha | 0.249 |
| AXIN2-14 | 22260072 | axin 2 | 0.255 |
| KRAS-11 | 18156006 | Kirsten rat sarcoma viral oncogene homolog | 0.258 |
| RAD51-22 | 11776794 | RAD51 homolog (RecA homolog, E. coli) (S. cerevisiae) | 0.262 |
| RAD51-21 | 11803252 | RAD51 homolog (RecA homolog, E. coli) (S. cerevisiae) | 0.271 |
| GSK3B-31 | 26170197 | glycogen synthase kinase 3 beta | 0.272 |
| RAD51-20 | 11815065 | RAD51 homolog (RecA homolog, E. coli) (S. cerevisiae) | 0.294 |
| KRAS-07 | 18144402 | Kirsten rat sarcoma viral oncogene homolog | 0.300 |
| MTHFR-03 | 6400424 | 5,10-methylenetetrahydrofolate reductase (NADPH) | 0.300 |
| GSK3B-41 | 26138327 | glycogen synthase kinase 3 beta | 0.313 |
| RXRA-01 | 229963 | retinoid X receptor, alpha | 0.314 |
| ENG-06 | 37900804 | endoglin | 0.333 |
| GSK3B-38 | 26130539 | glycogen synthase kinase 3 beta | 0.335 |
| KRAS-21 | 18150213 | Kirsten rat sarcoma viral oncogene homolog | 0.337 |
| GSK3B-18 | 26114476 | glycogen synthase kinase 3 beta | 0.339 |
| CCND1-01 | 326936 | cyclin D1 | 0.339 |
| NBN-04 | 1063053 | nibrin | 0.363 |
| TNKS-38 | 2020137 | tankyrase, TRF1-interacting ankyrin-related ADP-ribose polymerase | 0.370 |
| TERT-08 | 1286486 | telomerase reverse transcriptase | 0.377 |
| GSTM3-01 | 6187386 | glutathione S-transferase mu 3 (brain) | 0.388 |
| SLC6A3-10 | 1401412 | solute carrier family 6 (neurotransmitter transporter, dopamine), member 3 | 0.391 |
| MSH2-08 | 26445831 | mutS homolog 2, colon cancer, nonpolyposis type 1 (E. coli) | 0.393 |
| FOS-08 | 56747011 | v-fos FBJ murine osteosarcoma viral oncogene homolog | 0.403 |
| ABCA1-12 | 14907958 | ATP-binding cassette, sub-family A (ABC1), member 1 | 0.420 |
| TSG101-40 | 17290400 | tumor susceptibility gene 101 | 0.422 |
| GSK3B-17 | 26270293 | glycogen synthase kinase 3 beta | 0.428 |
| SLC23A2-25 | 4838896 | solute carrier family 23 (nucleobase transporters), member 2 | 0.430 |
| SCARB1-09 | 2697149 | scavenger receptor class B, member 1 | 0.431 |
| MSH3-02 | 30560387 | mutS homolog 2, colon cancer, nonpolyposis type 1 (E. coli) | 0.433 |
| TSG101-07 | 17323456 | tumor susceptibility gene 101 | 0.437 |
| TNKS-15 | 2039788 | tankyrase, TRF1-interacting ankyrin-related ADP-ribose polymerase | 0.437 |
| GSK3B-36 | 26287450 | glycogen synthase kinase 3 beta | 0.439 |
| WRN-04 | 1345428 | Werner syndrome | 0.441 |
| AURKA-06 | 6024840 | serine/threonine-protein kinase 6 | 0.445 |
| HTR1B-07 | 15993452 | 5-hydroxytryptamine (serotonin) receptor 1B | 0.447 |
| CCR2-02 | 46885 | chemokine (C-C motif) receptor 2 | 0.447 |
| CCR2-06 | 48119 | chemokine (C-C motif) receptor 2 | 0.455 |
| GSK3B-05 | 26073934 | glycogen synthase kinase 3 beta | 0.456 |
| ABCC2-02 | 20312341 | ATP-binding cassette, sub-family C (CFTR/MRP), member 2 | 0.458 |
| MSH3-07 | 30688158 | mutS homolog 2, colon cancer, nonpolyposis type 1 (E. coli) | 0.462 |
| RAD51-24 | 11797544 | RAD51 homolog (RecA homolog, E. coli) (S. cerevisiae) | 0.469 |
| AHR-19 | 16827388 | aryl hydrocarbon receptor | 0.470 |
| TERF1-02 | 25796065 | telomeric repeat binding factor (NIMA-interacting) 1 | 0.480 |
| TNKS-64 | 1948776 | tankyrase, TRF1-interacting ankyrin-related ADP-ribose polymerase | 0.485 |
| CASR-09 | 28496392 | calcium-sensing receptor | 0.489 |
| CDC25B-06 | 3727496 | cell division cycle 25 homolog B | 0.490 |
| AXIN2-12 | 22257691 | axin 2 | 0.491 |
| TSG101-28 | 17292873 | tumor susceptibility gene 101 | 0.498 |
| VCAM1-38 | 71162092 | vascular cell adhesion molecule 1 | 0.501 |
| KRAS-16 | 18150038 | Kirsten rat sarcoma viral oncogene homolog | 0.504 |
| TP53-14 | 7168801 | tumor protein p53 | 0.504 |
| WDR79-08 | 2287498 | WD repeat domain 79 | 0.505 |
| CGA-02 | 25615430 | glycoprotein hormones, alpha polypeptide | 0.505 |
| GSK3B-25 | 26122393 | glycogen synthase kinase 3 beta | 0.507 |
| GSK3B-11 | 26299282 | glycogen synthase kinase 3 beta | 0.508 |
| KRAS-17 | 18141160 | Kirsten rat sarcoma viral oncogene homolog | 0.509 |
| IGF2R-04 | 2781750 | insulin-like growth factor 2 receptor | 0.509 |

**Supplementary Table 1** Top 100 SNPs selected by Evaporative Cooling (EC) as most relevant to smallpox vaccine-associated adverse events. SNPs sorted from best to worst EC score. SNPs are named according to their SNP500Cancer id (<http://snp500cancer.nci.nih.gov/>) in the first column and by dbSNP number (build 129) in the second column.
