# Supplementary material for: Capturing the Spectrum of Interaction Effects in Genetic Association Studies by Simulated Evaporative Cooling Network Analysis
Source: PLoS Genet. 2009 Mar 20;5(3):e1000432. doi: 10.1371/journal.pgen.1000432 (PMC2653647; doi:10.1371/journal.pgen.1000432)
Supplement: Table S2 — Pairs of SNPs ranked by their total joint information gain (last column), which is the sum of IG1, IG2, and pair-wise interaction information (II). IG1 (respectively, IG2) is the information gained about the phenotype variable when SNP interaction partner 1 (respectively, 2) is measured by itself. II (column 5) is the information gained about the phenotype when considering the SNP partners 1 and 2 jointly over what would be expected by their independent information gains. II is used to specify edge properties in Figure 3. Information gains are given as percentages, where perfect correlation with the phenotype is 100%. SNPs are named according to their SNP500Cancer id (http://snp500cancer.nci.nih.gov/). (0.25 MB DOC) [file pgen.1000432.s002.doc]

| **SNP Partner 1** | **IG1 (%)** | **SNP Partner 2** | **IG2 (%)** | **II (%)** | **Total Gain (%)** |
| --- | --- | --- | --- | --- | --- |
| GSK3B_01 | 16.37 | LIPC_08 | 0.45 | 12.14 | 28.97 |
| RXRA_03 | 4.70 | KRAS_02 | 13.10 | 10.86 | 28.66 |
| RXRA_03 | 4.70 | GSK3B_01 | 16.37 | 6.95 | 28.02 |
| KRAS_02 | 13.10 | LIPC_08 | 0.45 | 14.41 | 27.96 |
| GSK3B_01 | 16.37 | EXO1_02 | 0.03 | 11.18 | 27.58 |
| SCUBE2_02 | 2.53 | GSK3B_01 | 16.37 | 8.67 | 27.57 |
| IL2_03 | 1.95 | GSK3B_01 | 16.37 | 8.98 | 27.31 |
| SLC6A3_05 | 4.19 | GSK3B_01 | 16.37 | 6.22 | 26.78 |
| SLC6A3_10 | 4.82 | ARNT_23 | 9.95 | 11.31 | 26.07 |
| TNKS_20 | 2.00 | GSK3B_01 | 16.37 | 7.69 | 26.06 |
| IL8RA_04 | 1.30 | GSK3B_01 | 16.37 | 7.93 | 25.61 |
| ABCA1_12 | 2.72 | GSK3B_01 | 16.37 | 6.04 | 25.14 |
| KRAS_02 | 13.10 | EXO1_02 | 0.03 | 11.46 | 24.59 |
| RAD51_20 | 1.35 | KRAS_02 | 13.10 | 9.34 | 23.78 |
| AURKA_06 | 0.46 | GSK3B_01 | 16.37 | 6.74 | 23.57 |
| ESR1_13 | 2.17 | KRAS_02 | 13.10 | 8.18 | 23.45 |
| GSK3B_01 | 16.37 | CASR_09 | 0.24 | 6.15 | 22.76 |
| KRAS_02 | 13.10 | HTR1B_07 | 0.12 | 8.96 | 22.18 |
| KRAS_02 | 13.10 | ABCA1_12 | 2.72 | 6.26 | 22.09 |
| AXIN2_14 | 8.44 | LIPC_08 | 0.45 | 12.63 | 21.52 |
| ALOX5_15 | 2.93 | AHR_17 | 8.41 | 10.01 | 21.35 |
| IL2_03 | 1.95 | KRAS_02 | 13.10 | 6.11 | 21.15 |
| LIPC_08 | 0.45 | ARNT_23 | 9.95 | 10.70 | 21.10 |
| SLC6A3_05 | 4.19 | AHR_17 | 8.41 | 8.37 | 20.96 |
| AXIN2_14 | 8.44 | SLC6A3_05 | 4.19 | 8.19 | 20.82 |
| XPA_02 | 2.43 | AXIN2_14 | 8.44 | 9.70 | 20.57 |
| KRAS_02 | 13.10 | GSK3B_01 | 16.37 | -9.11 | 20.36 |
| KRAS_02 | 13.10 | MTHFR_03 | 0.15 | 6.94 | 20.19 |
| AXIN2_12 | 4.81 | MTHFR_02 | 6.90 | 8.44 | 20.15 |
| AXIN2_14 | 8.44 | EXO1_02 | 0.03 | 11.57 | 20.05 |
| XPA_02 | 2.43 | GSK3B_11 | 5.92 | 11.45 | 19.80 |
| RXRA_03 | 4.70 | AHR_17 | 8.41 | 6.65 | 19.75 |
| ESR1_13 | 2.17 | ARNT_23 | 9.95 | 7.22 | 19.34 |
| EXO1_02 | 0.03 | ARNT_23 | 9.95 | 9.31 | 19.29 |
| IL2_03 | 1.95 | ARNT_23 | 9.95 | 7.31 | 19.21 |
| GSK3B_01 | 16.37 | ARNT_23 | 9.95 | -7.17 | 19.15 |
| OPRD1_03 | 3.84 | MTHFR_02 | 6.90 | 8.28 | 19.02 |
| XPA_02 | 2.43 | ARNT_23 | 9.95 | 6.38 | 18.75 |
| SLC6A3_05 | 4.19 | GSK3B_11 | 5.92 | 8.24 | 18.35 |
| MSH2_08 | 7.79 | LIPC_08 | 0.45 | 9.91 | 18.16 |
| OPRD1_03 | 3.84 | CBR3_01 | 5.16 | 8.83 | 17.83 |
| MSH2_08 | 7.79 | CDC25B_06 | 2.94 | 6.99 | 17.72 |
| SLC23A2_25 | 2.36 | GSK3B_11 | 5.92 | 9.33 | 17.60 |
| KRAS_17 | 5.00 | SLC6A3_05 | 4.19 | 8.33 | 17.51 |
| OPRD1_03 | 3.84 | TP53_14 | 5.50 | 8.16 | 17.50 |
| EXO1_02 | 0.03 | AHR_17 | 8.41 | 8.93 | 17.37 |
| TNKS_64 | 6.14 | SLC6A3_05 | 4.19 | 6.98 | 17.31 |
| MSH2_08 | 7.79 | EXO1_02 | 0.03 | 9.45 | 17.28 |
| CASR_09 | 0.24 | ARNT_23 | 9.95 | 7.04 | 17.22 |
| ESR1_13 | 2.17 | RXRA_01 | 4.22 | 10.81 | 17.20 |
| MSH2_08 | 7.79 | SLC6A3_14 | 3.04 | 6.31 | 17.14 |
| AXIN2_12 | 4.81 | EXO1_02 | 0.03 | 12.21 | 17.06 |
| CD4_03 | 7.29 | GSTM3_01 | 1.58 | 8.16 | 17.02 |
| AXIN2_14 | 8.44 | IL2_03 | 1.95 | 6.59 | 16.98 |
| AHR_17 | 8.41 | LIPC_08 | 0.45 | 8.03 | 16.89 |
| AXIN2_14 | 8.44 | GSK3B_01 | 16.37 | -8.05 | 16.77 |
| HTR1B_07 | 0.12 | ARNT_23 | 9.95 | 6.69 | 16.75 |
| GSK3B_17 | 0.90 | AHR_17 | 8.41 | 7.36 | 16.67 |
| MSH2_08 | 7.79 | IL2_03 | 1.95 | 6.84 | 16.58 |
| KRAS_17 | 5.00 | LIPC_08 | 0.45 | 11.13 | 16.58 |
| GSK3B_11 | 5.92 | LIPC_08 | 0.45 | 10.09 | 16.47 |
| SLC6A3_05 | 4.19 | CCR2_02 | 2.98 | 9.09 | 16.26 |
| RAD51_20 | 1.35 | AXIN2_14 | 8.44 | 6.34 | 16.13 |
| CD4_03 | 7.29 | ABCA1_12 | 2.72 | 6.03 | 16.05 |
| RXRA_01 | 4.22 | VCAM1_38 | 0.35 | 11.38 | 15.94 |
| CCND1_01 | 2.30 | TNKS_15 | 1.63 | 11.96 | 15.89 |
| TERT_08 | 0.24 | MSH2_08 | 7.79 | 7.62 | 15.65 |
| RXRA_03 | 4.70 | SLC6A3_05 | 4.19 | 6.76 | 15.65 |
| AXIN2_12 | 4.81 | SLC6A3_05 | 4.19 | 6.57 | 15.58 |
| ESR1_13 | 2.17 | SLC6A3_05 | 4.19 | 8.74 | 15.10 |
| KRAS_02 | 13.10 | AHR_17 | 8.41 | -6.46 | 15.05 |
| XPA_02 | 2.43 | AXIN2_12 | 4.81 | 7.77 | 15.01 |
| CYBB_12 | 7.38 | ENG_06 | 0.06 | 7.57 | 15.01 |
| RAD51_20 | 1.35 | MTHFR_02 | 6.90 | 6.71 | 14.96 |
| KRAS_02 | 13.10 | ARNT_23 | 9.95 | -8.10 | 14.94 |
| AHR_17 | 8.41 | ENG_06 | 0.06 | 6.30 | 14.77 |
| AXIN2_14 | 8.44 | KRAS_02 | 13.10 | -6.88 | 14.66 |
| KRAS_17 | 5.00 | IL2_03 | 1.95 | 7.57 | 14.51 |
| KRAS_17 | 5.00 | EXO1_02 | 0.03 | 9.39 | 14.41 |
| OPRD1_03 | 3.84 | SLC6A3_05 | 4.19 | 6.30 | 14.33 |
| AHR_19 | 3.12 | SLC6A3_05 | 4.19 | 6.98 | 14.28 |
| IL4_01 | 2.69 | RAD51_24 | 0.82 | 10.74 | 14.24 |
| GSK3B_17 | 0.90 | LIPC_08 | 0.45 | 12.78 | 14.14 |
| AURKA_06 | 0.46 | CYBB_12 | 7.38 | 6.14 | 13.98 |
| RXRA_03 | 4.70 | SCUBE2_02 | 2.53 | 6.72 | 13.95 |
| RXRA_03 | 4.70 | CDC25B_06 | 2.94 | 6.29 | 13.92 |
| SLC6A3_05 | 4.19 | ENG_06 | 0.06 | 9.65 | 13.89 |
| IL4_01 | 2.69 | NBN_04 | 5.09 | 6.01 | 13.80 |
| ALOX5_15 | 2.93 | CASR_06 | 4.21 | 6.50 | 13.64 |
| XPA_02 | 2.43 | KRAS_17 | 5.00 | 6.16 | 13.58 |
| RXRA_03 | 4.70 | CCND1_01 | 2.30 | 6.45 | 13.45 |
| AXIN2_12 | 4.81 | GSTM3_01 | 1.58 | 7.03 | 13.43 |
| CCND1_01 | 2.30 | KRAS_17 | 5.00 | 6.13 | 13.43 |
| ABCA1_12 | 2.72 | SLC6A3_05 | 4.19 | 6.30 | 13.21 |
| TNKS_64 | 6.14 | CASR_09 | 0.24 | 6.61 | 12.99 |
| CGA_02 | 0.98 | CCR2_02 | 2.98 | 9.01 | 12.97 |
| TNKS_64 | 6.14 | EXO1_02 | 0.03 | 6.79 | 12.96 |
| TERT_08 | 0.24 | SLC6A3_05 | 4.19 | 8.51 | 12.94 |
| ABCC2_02 | 1.96 | RXRA_01 | 4.22 | 6.76 | 12.94 |
| GSK3B_17 | 0.90 | AXIN2_12 | 4.81 | 7.20 | 12.91 |
| GSK3B_17 | 0.90 | IL2_03 | 1.95 | 10.05 | 12.90 |
| ESR1_13 | 2.17 | CASR_06 | 4.21 | 6.46 | 12.84 |
| GSK3B_17 | 0.90 | SLC6A3_05 | 4.19 | 7.73 | 12.82 |
| OPRD1_03 | 3.84 | ABCA1_12 | 2.72 | 6.18 | 12.75 |
| GSK3B_17 | 0.90 | CCR2_02 | 2.98 | 8.83 | 12.71 |
| TERT_08 | 0.24 | CBR3_01 | 5.16 | 7.27 | 12.67 |
| TNKS_64 | 6.14 | LIPC_08 | 0.45 | 6.00 | 12.60 |
| RXRA_03 | 4.70 | TNKS_15 | 1.63 | 6.15 | 12.47 |
| TP53_14 | 5.50 | TERT_08 | 0.24 | 6.68 | 12.42 |
| NBN_04 | 5.09 | RAD51_24 | 0.82 | 6.49 | 12.40 |
| OPRD1_03 | 3.84 | RAD51_20 | 1.35 | 7.19 | 12.38 |
| ABCC2_02 | 1.96 | SLC6A3_05 | 4.19 | 6.21 | 12.35 |
| CGA_02 | 0.98 | CASR_06 | 4.21 | 7.15 | 12.34 |
| SCUBE2_02 | 2.53 | IGF2R_04 | 3.45 | 6.35 | 12.33 |
| GSK3B_17 | 0.90 | SCUBE2_02 | 2.53 | 8.86 | 12.29 |
| SLC6A3_14 | 3.04 | CCR2_02 | 2.98 | 6.19 | 12.22 |
| CGA_02 | 0.98 | LIPC_08 | 0.45 | 10.74 | 12.17 |
| AURKA_06 | 0.46 | AXIN2_12 | 4.81 | 6.75 | 12.03 |
| SCUBE2_02 | 2.53 | TSG101_40 | 3.01 | 6.35 | 11.89 |
| ALOX5_15 | 2.93 | CDC25B_06 | 2.94 | 6.02 | 11.89 |
| OPRD1_03 | 3.84 | BLM_02 | 1.65 | 6.34 | 11.83 |
| OPRD1_03 | 3.84 | IL2_03 | 1.95 | 6.01 | 11.80 |
| SCUBE2_02 | 2.53 | CCR2_02 | 2.98 | 6.27 | 11.79 |
| XPA_02 | 2.43 | AHR_19 | 3.12 | 6.15 | 11.70 |
| AXIN2_14 | 8.44 | ARNT_23 | 9.95 | -6.75 | 11.64 |
| AXIN2_12 | 4.81 | LIPC_08 | 0.45 | 6.17 | 11.44 |
| RXRA_01 | 4.22 | ENG_06 | 0.06 | 7.11 | 11.39 |
| TERT_08 | 0.24 | KRAS_17 | 5.00 | 6.14 | 11.38 |
| GSK3B_17 | 0.90 | RXRA_01 | 4.22 | 6.07 | 11.19 |
| TNKS_20 | 2.00 | CCND1_01 | 2.30 | 6.78 | 11.08 |
| ABCA1_12 | 2.72 | CASR_09 | 0.24 | 7.99 | 10.96 |
| SLC6A3_14 | 3.04 | LIPC_08 | 0.45 | 6.85 | 10.34 |
| GSK3B_17 | 0.90 | CCND1_01 | 2.30 | 6.96 | 10.16 |
| CCND1_01 | 2.30 | EXO1_02 | 0.03 | 7.74 | 10.08 |
| ENG_06 | 0.06 | CDC25B_06 | 2.94 | 6.90 | 9.89 |
| GSK3B_17 | 0.90 | IL4_01 | 2.69 | 6.18 | 9.76 |
| TNKS_15 | 1.63 | IL2_03 | 1.95 | 6.14 | 9.71 |
| GSK3B_17 | 0.90 | ENG_06 | 0.06 | 8.70 | 9.66 |
| AURKA_06 | 0.46 | XPA_02 | 2.43 | 6.72 | 9.61 |
| RAD51_20 | 1.35 | GSK3B_17 | 0.90 | 7.27 | 9.52 |
| ABCC2_02 | 1.96 | VCAM1_38 | 0.35 | 7.18 | 9.48 |
| HTR1B_07 | 0.12 | LIPC_08 | 0.45 | 8.87 | 9.45 |
| SLC23A2_25 | 2.36 | CGA_02 | 0.98 | 6.11 | 9.44 |
| XPA_02 | 2.43 | GSK3B_17 | 0.90 | 6.01 | 9.34 |
| GSK3B_17 | 0.90 | ESR1_13 | 2.17 | 6.15 | 9.23 |
| GSK3B_17 | 0.90 | IL8RA_04 | 1.30 | 7.00 | 9.20 |
| HTR1B_07 | 0.12 | TSG101_40 | 3.01 | 6.05 | 9.18 |
| CCND1_01 | 2.30 | ENG_06 | 0.06 | 6.82 | 9.18 |
| IL2_03 | 1.95 | ENG_06 | 0.06 | 7.07 | 9.07 |
| TNKS_20 | 2.00 | GSK3B_17 | 0.90 | 6.04 | 8.94 |
| TERT_08 | 0.24 | TNKS_15 | 1.63 | 7.04 | 8.90 |
| GSK3B_17 | 0.90 | HTR1B_07 | 0.12 | 7.84 | 8.86 |
| GSK3B_17 | 0.90 | CASR_09 | 0.24 | 7.68 | 8.82 |
| TNKS_20 | 2.00 | EXO1_02 | 0.03 | 6.43 | 8.46 |
| CGA_02 | 0.98 | HTR1B_07 | 0.12 | 6.93 | 8.03 |
| GSK3B_17 | 0.90 | VCAM1_38 | 0.35 | 6.64 | 7.89 |
| RAD51_20 | 1.35 | ENG_06 | 0.06 | 6.24 | 7.65 |
| GSK3B_17 | 0.90 | EXO1_02 | 0.03 | 6.68 | 7.61 |
| RAD51_24 | 0.82 | LIPC_08 | 0.45 | 6.00 | 7.27 |
| AURKA_06 | 0.46 | ENG_06 | 0.06 | 6.20 | 6.72 |

**Supplementary Table 2.** Pairs of SNPs ranked by their total joint information gain (last column), which is the sum of IG1, IG2, and pair-wise interaction information (II). IG1 (respectively, IG2) is the information gained about the phenotype variable when SNP interaction partner 1 (respectively, 2) is measured by itself. II (column 5) is the information gained about the phenotype when considering the SNP partners 1 and 2 jointly over what would be expected by their independent information gains. II is used to specify edge properties in Fig. 3. Information gains are given as percentages, where perfect correlation with the phenotype is 100%. SNPs are named according to their SNP500Cancer id (<http://snp500cancer.nci.nih.gov/>).
