# Supplementary material for: Capturing the Spectrum of Interaction Effects in Genetic Association Studies by Simulated Evaporative Cooling Network Analysis
Source: PLoS Genet. 2009 Mar 20;5(3):e1000432. doi: 10.1371/journal.pgen.1000432 (PMC2653647; doi:10.1371/journal.pgen.1000432)
Supplement: Table S3 — Degree distribution of nodes in the genetic-association interaction network (GAIN) in Figure 3 for the smallpox vaccine-associated adverse event phenotype. SNPs are sorted by the degree (number of connections) of each SNP node in the network. SNPs are named according to their SNP500Cancer id (http://snp500cancer.nci.nih.gov/). dbSNP numbers can be found in Table S1. (0.07 MB DOC) [file pgen.1000432.s003.doc]

| **Node** | **Degree** |
| --- | --- |
| GSK3B_17 | 20 |
| SLC6A3_05 | 17 |
| LIPC_08 | 16 |
| GSK3B_01 | 14 |
| KRAS_02 | 13 |
| EXO1_02 | 13 |
| ENG_06 | 11 |
| ARNT_23 | 11 |
| IL2_03 | 10 |
| AXIN2_14 | 9 |
| OPRD1_03 | 8 |
| XPA_02 | 8 |
| RXRA_03 | 8 |
| AXIN2_12 | 8 |
| AHR_17 | 8 |
| RAD51_20 | 7 |
| CCND1_01 | 7 |
| KRAS_17 | 7 |
| TERT_08 | 6 |
| SCUBE2_02 | 6 |
| MSH2_08 | 6 |
| ESR1_13 | 6 |
| ABCA1_12 | 6 |
| HTR1B_07 | 6 |
| GSK3B_25 | 5 |
| AURKA_06 | 5 |
| CGA_02 | 5 |
| RXRA_01 | 5 |
| CCR2_02 | 5 |
| CASR_09 | 5 |
| TNKS_64 | 4 |
| TNKS_20 | 4 |
| ALOX5_15 | 4 |
| TNKS_15 | 4 |
| GSK3B_11 | 4 |
| CDC25B_06 | 4 |
| IL4_01 | 3 |
| ABCC2_02 | 3 |
| MTHFR_02 | 3 |
| SLC6A3_14 | 3 |
| RAD51_24 | 3 |
| CASR_06 | 3 |
| VCAM1_38 | 3 |
| SLC23A2_25 | 2 |
| TP53_14 | 2 |
| IL8RA_04 | 2 |
| CD4_03 | 2 |
| CBR3_01 | 2 |
| AHR_19 | 2 |
| CYBB_12 | 2 |
| NBN_04 | 2 |
| TSG101_40 | 2 |
| GSTM3_01 | 2 |
| BLM_02 | 1 |
| IGF2R_04 | 1 |
| SLC6A3_10 | 1 |
| MTHFR_03 | 1 |

**Supplementary Table 3.**  Degree distribution of nodes in the genetic-association interaction network (GAIN) in Fig. 3 for the smallpox vaccine-associated adverse event phenotype. SNPs are sorted by the degree (number of connections) of each SNP node in the network. SNPs are named according to their SNP500Cancer id (<http://snp500cancer.nci.nih.gov/>). dbSNP numbers can be found in Supplementary Table 1.
